# High sensitivity of middle-wavelength infrared photodetectors based on an individual InSb nanowire

**DOI:** 10.1186/1556-276X-8-327

**Published:** 2013-07-18

**Authors:** Cheng-Hsiang Kuo, Jyh-Ming Wu, Su-Jien Lin, Wen-Chih Chang

**Affiliations:** 1 Department of Materials Science & Engineering, National Tsing Hua University, No. 101, Sec. 2, Kuang-Fu Rd, Hsinchu 30013, Taiwan

**Keywords:** Electrochemical method, InSb nanowires, Middle-infrared, Photodetectors, Metal–semiconductor-metal structure

## Abstract

Single-crystal indium antimony (InSb) nanowire was fabricated into middle-infrared photodetectors based on a metal–semiconductor-metal (M-S-M) structure. The InSb nanowires were synthesized using an electrochemical method at room temperature. The characteristics of the FET reveal an electron concentration of 3.6 × 10^17^ cm^−3^ and an electron mobility of 215.25 cm^2^ V^−1^ s^−1^. The photodetectors exhibit good photoconductive performance, excellent stability, reproducibility, superior responsivity (8.4 × 10^4^ A W^−1^), and quantum efficiency (1.96 × 10^6^%). These superior properties are attributed to the high surface-to-volume ratio and single-crystal 1D nanostructure of photodetectors that significantly reduce the scattering, trapping, and the transit time between the electrodes during the transport process. Furthermore, the M-S-M structure can effectively enhance space charge effect by the formation of the Schottky contacts, which significantly assists with the electron injection and photocurrent gain.

## Background

One-dimensional (1D) nanostructure materials have received considerable attention because of their importance in potential applications in electronics and photoelectric nanodevices [1]. With high surface-to-volume ratio and Debye lengths comparable to nanomaterial's diameters, the electronic and photoelectric properties of 1D nanostructure are strongly affected by the surface effect via chemisorption (oxygen adsorption) and native surface defects [2-4]. Thus, 1D nanostructure exhibits a superior sensitivity to light and chemical molecules compared to the thin film and bulk. Due to these properties, electronic devices fabricated using 1D nanostructure have been extensively adapted in photodetectors [5], gas sensors [6], and dye-sensitized solar cells [7], respectively. Of these application fields, photodetectors or switches based on semiconductor materials have been the focus of considerable attention in recent years because of their high sensitivity and high quantum efficiency. Furthermore, the different energy band gaps imply that photodetectors can be applied flexibly on various wavelengths. To date, photodetectors based on 1D semiconductor nanostructures, such as SnO_2_ nanowires [8], ZnO nanowires [9], ZnSe nanobelts [10], CdS nanoribbons [11], and CuO nanowires [12], have been reported. These 1D nanostructure photodetectors exhibit outstanding performance; however, the detection range that has been investigated so far falls primarily between the infrared and ultraviolet region. In fact, 1D nanostructure photodetectors of the mid- to long-wavelength infrared (IR) region have seldom been reported because only a few other materials can be used in this region.

Indium antimony (InSb), one of the III-V compounds with a face-centered cubic structure of the zincblende type, is a useful material for producing mid- to long-wavelength IR photodetectors because of the smallest band gap (*E*_g_ = 0.17 eV, at 300 K). In addition, owing to the small effective mass (m*_e_ = 0.014 *m*_o_) and the ballistic length (up to 0.7 μm at 300 K), InSb has an extremely high carrier mobility (i.e., electron mobility of 77,000 cm^2^V^-1^s^-1^) [13]. Therefore, InSb is a highly promising material for device applications involving high-speed-response electronic nanodevices, optical communication devices, and optical detectors [13,14]. Owing to the aforementioned unique characteristics, now, many groups use different synthesis methods to produce InSb nanowires, i.e., chemical beam epitaxy [15], chemical vapor deposition [16], and pulsed laser deposition (PLD) [17]. Meanwhile, the electrical transport characteristics are also widely investigated [18,19]. However, only few groups study on the IR detectors, particularly on the mid- to long-wavelength region [20,21]. This work shows that InSb nanowires can be successfully synthesized at room temperature by applying electrochemical method with an anodic aluminum oxide (AAO) template. The synthesizing process was simple, fast, and straightforward in fabricating large-area InSb nanowires at low temperature compared to other thermal reactive processes. Moreover, individual InSb nanowires based on a metal–semiconductor-metal (M-S-M) structure were fabricated into the photodetectors. It shows high sensitivity, good stability, reproducibility, and response speed after illumination with middle-infrared (M-IR; 5.5 μm) light. Furthermore, a systematic study of the photoresponse was performed, which revealed a clear dependence of the photocurrent, carrier lifetime, and quantum efficiency on the light intensity, defect, and M-S-M structure.

## Methods

InSb nanowires were synthesized using the electrochemical method. A gold (Au) film coated on an AAO (Whatman®, GE Healthcare, Maidstone, UK) membrane was used as a conductive layer to grow the nanowires. The pore diameter of the AAO membrane was approximately 200 nm. The electrolyte consisted of 0.15 M InCl_3_, 0.1 M SbCl_3_, 0.36 M C_6_H_8_O_7_·H_2_O, and 0.17 M KCl. The solvent of the electrolyte was distilled water. A typical three-electrode electrochemical cell was used during the InSb electrodeposition. The Au film on the AAO membrane was regarded as the working electrode. A platinum wire and an Ag/AgCl electrode were subsequently applied as the counter electrode and the reference electrode, respectively. The deposition time was controlled at 40 min in conditions of a deposition potential of −1.5 V, in contrast to the Ag/AgCl reference electrode at room temperature. Following the deposition, the sample was removed from the AAO membrane with a 5 wt % NaOH solution and then washed five times with distilled water.

The as-prepared nanowires were examined using field emission scanning electron microscope (FESEM; operated at 10 kV; HITACHI S-4800, Chiyoda-ku, Japan), a desktop X-ray diffractometer (D2 Phaser, Bruker, Madison, WI, USA), a high-resolution transmission electron microscope (HRTEM; operated at 200 kV, JEM-2100F, JEOL Ltd., Tokyo, Japan) with energy-dispersive X-ray spectroscope (EDX), and an X-ray photoelectron spectroscope system (PHI600 system, PerkinElmer, Waltham, MA, USA). Furthermore, the transport property was evaluated using the InSb nanowires further fabricated into a field-effect transistor (FET). The synthesized InSb nanowires were dispersed uniformly in ethanol and dropped on a SiO_2_/p-Si substrate. The Si substrate was applied as a back-gate. After drying out the suspension, the Ti/Cu (20/120 nm) electrodes were deposited on the two ends of the nanowire through photolithograph, e-beam evaporation, and lift-off processes. Additionally, the InSb nanowire-based M-S-M structure photodetectors were fabricated through a microfabrication process and focused ion beam (FIB) technique. Here, the pattern of Ti/Au (20/120 nm) electrode was fabricated using standard lithographic methods on a SiO_2_/Si substrate. The synthesized InSb nanowires were transferred onto a SiO_2_/Si substrate with pre-patterned Ti/Au electrodes. Subsequently, the FIB instrument (Dual-Beam Helios 600i, FEI, Shanghai, China) was used to deposit Pt, which connects the wires between the Ti/Au electrodes. Finally, The Pt-InSb-Pt (M-S-M) photodetector structure of back-to-back Schottky contacts was obtained. To evaluate the M-S-M photodetectors, a M-IR light at a 5.5-μm wavelength was used as an excitation light source. The transport and photosensitivity properties were analyzed using the semiconductor characterization system (4200-SCS, Keithley Instruments Inc., Cleveland, OH, USA) at room temperature.

## Results and discussion

**Figure 1:**
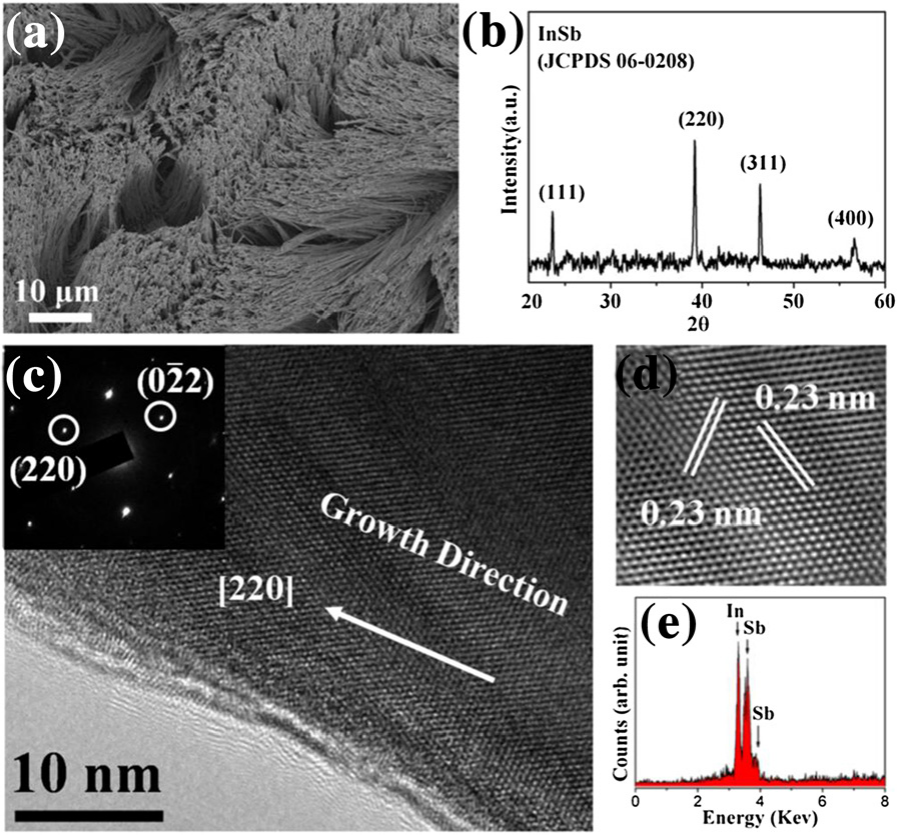
**SEM image**, **XRD pattern**, **TEM and HRTEM images**, **and EDX spectrum of synthesized InSb nanowires**. **(a)** SEM image shows the well-aligned and dense InSb, in which the image reveals the diameter (200 nm) of the InSb nanowires. **(b)** XRD pattern of the synthesized InSb nanowires. **(c)** An HRTEM image of InSb nanowires reveals the preferred growth orientation being along [220]. The inset is a selected area electron diffraction (SAED) image. **(d)** The enlarged HRTEM image shows the clear lattice spacing of atomic planes. **(e)** EDX spectrum shows the composition of the synthesized InSb nanowire.

The typical FESEM image, shown in Figure 1a, indicated that the InSb nanowires are abundant, well-aligned, and uniformly distributed on the Au layer, with diameters of approximately 200 nm, which correspond to the pore size of the AAO membrane. Their length reached up to several tens of micrometers. Figure 1b shows the XRD pattern of the characterized crystalline structure of synthesized products. The diffraction peaks could be indexed to the zincblende structure of InSb (JCPDS 06–0208) with lattice constants of 0.64 nm. The pattern presented no In and Sb peaks, except for the high-purity InSb structure.

In the analysis, the defect structure and the crystallinity of the synthesized nanowires were more closely examined using HRTEM. Figure 1c shows an HRTEM image of a single InSb nanowire and a corresponding selected area electron diffraction (SAED) pattern from the nanowire as the inset. Both the SAED pattern and the HRTEM image verify that the synthesized InSb nanowires have a single-crystal zincblende structure. The SAED pattern indicates that [220] is the preferred growth orientation of InSb nanowires, which coincides with the XRD result. The enlarged HRTEM image in Figure 1d revealed a clear lattice spacing of atomic planes of approximately 0.23 nm corresponding to the {220} plane of InSb. According to the EDX spectrum, the composition of the synthesized nanowires was only In and Sb. The composition ratio of In/Sb was approximately 1:1, as shown in Figure 1e. The InSb nanowires were formed using the electrochemical method at room temperature. Both InCl_3_ and SbCl_3_ provided metal ion sources to synthesize the InSb nanowires. Because of the difference in the deposition potential of In and Sb, C_6_H_8_O_7_·H_2_O was used to enable the deposition potentials of In and Sb to approach each other. In addition, the KCl concentration controlled the deposition rate of In and Sb to achieve a precipitation ratio of 1:1. Moreover, the precipitation of In and Sb could spontaneously form InSb (*Δ*G_300K_ < 0) at room temperature (as shown in Equation ( 1)). The equation for the formation reaction can be expressed as:

(1) $${\mathrm{In}}_{\left(s\right)}{+\mathrm{Sb}}_{\left(s\right)}{=\mathrm{InSb}}_{\left(s\right)}{,\mathrm{\Delta G}}_{300K}≒‒26.4\;\mathrm{kJ}/\mathrm{mole}$$

The negative Gibbs energy (*Δ*G) implies that the formation reaction is spontaneous. Equation (1) demonstrates the feasibility of applying the electrochemical method to synthesize the InSb nanowires at room temperature.

**Figure 2:**
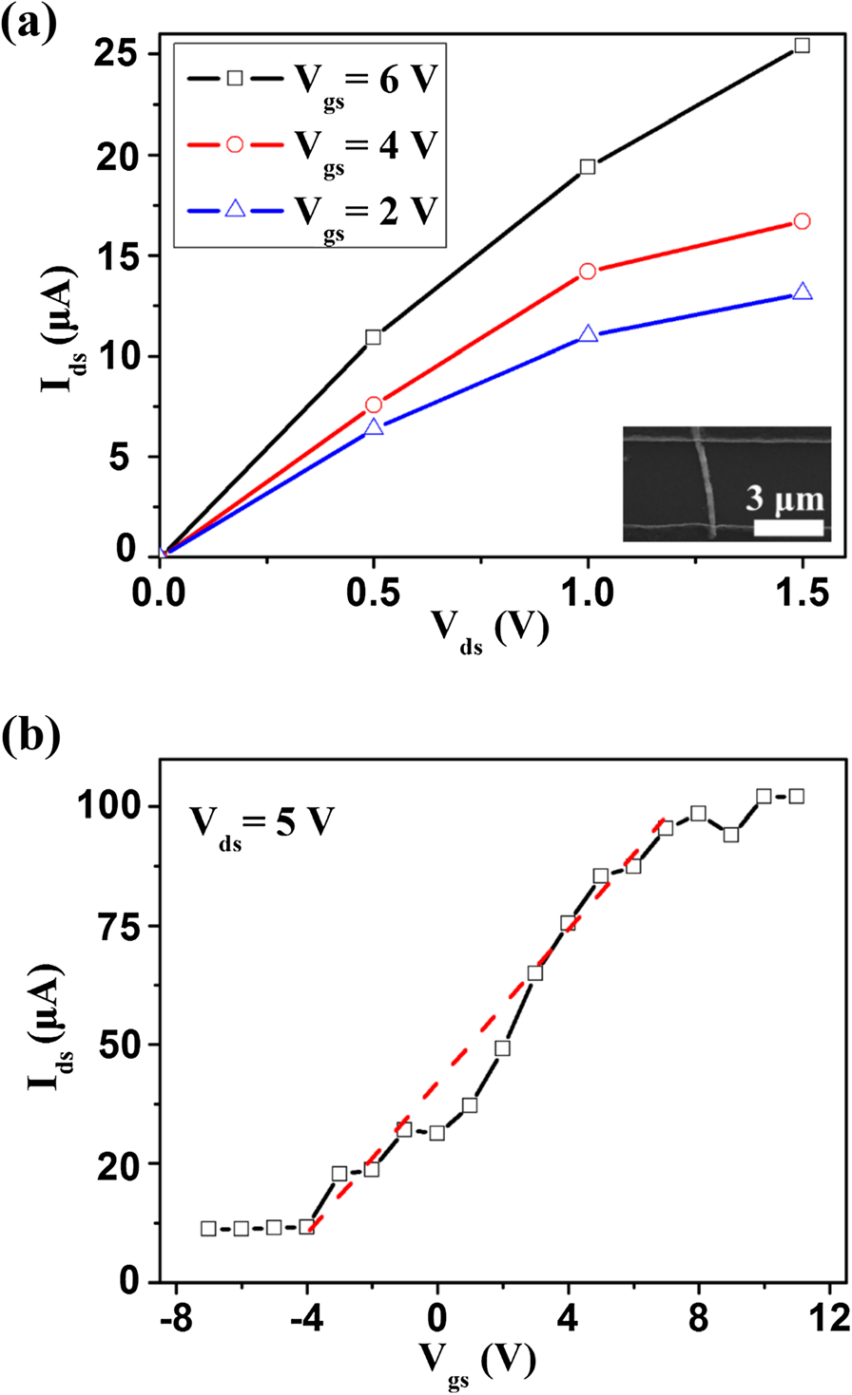
**The characteristics of the field-effect transistor based on an individual InSb nanowire. (a)***I*_ds_ versus *V*_ds_ characteristic curve under different gate voltage. The inset shows the SEM image of FET based on a single InSb nanowire. **(b)***I*_ds_ versus *V*_gs_ characteristic curve at *V*_ds_ = 5 V. The carrier concentration of 3.6 × 10^17^ cm^−3^ and mobility of 215.25 cm^2^ V^−1^ s^−1^ are obtained.

To evaluate the basic electrical transport characteristics of the as-prepared InSb nanowire, a FET was fabricated. Figure 2a shows the *I*_ds_ versus *V*_ds_ curve of the single InSb nanowire under various *V*_gs_ (gate bias) from 2 to 6 V. The *I*_ds_ versus *V*_ds_ curve of the InSb nanowire revealed a pronounced n-type semiconductor property, in which the current of the nanowire increases with an increasing gate bias. The n-type conductivity might have originated from the Sb vacancies in the InSb nanowires [22-24]. The Sb vacancy may derive from the surface defects, as reported in our previous work [25]. Additionally, other semiconductor-related studies described the vacancy-induced n-type conductivity in 1D nanoscale [26,27]. The inset revealed the SEM image of the single InSb nanowire connected to Cu electrodes. Figure 2b shows that *I*_ds_ is dependent on *V*_gs_ at *V*_ds_ as 5 V. The *I*_ds_ increased when *V*_gs_ increased from −7 to 11 V; in addition, the *I*_on_/*I*_off_ ratio was only approximately 8.9. The channel transconductance could be deduced based on the linear region from −4 to 7 V. Correspondingly, the electron mobility (*μ*) of the InSb nanowire could be estimated using the following equation [28]:

(2) $$\mu =g_{m}L^{2}∕{\mathrm{CV}}_{\mathrm{ds}}$$

where g_m_ is the channel transconductance of FET g_m_ = ∂ I_ds_ / ∂ V_gs_. C is the nanowire capacitance, and L is the nanowire length between the electrodes. The capacitance of the nanowire can be regarded as $C=2{\pi \epsilon }_{0}\epsilon_{{\mathrm{SiO}}_{2}}L/\ln\left(4h/d\right)$, where $\epsilon_{{\mathrm{SiO}}_{2}}$ is the dielectric constant of SiO_2_ (approximately 3.9), ϵ_0_ is the vacuum permittivity, *h* is the thickness of SiO_2_ (120 nm), and *d* is the average radius of the InSb nanowires. These equations show that the calculation of the *μ* is 215.25 cm^2^ V^−1^ s^−1^ at *V*_ds_ = 5 V. The value is about two times higher than the reported value of PLD fabricated InSb nanowires [17]. However, the value is much smaller than those of the bulk and other reported InSb nanowires [29,30]. The possible reasons are attributed to the scattering and trapping of electrons, and high contact resistance [31,32]. The trapping of electrons in the trap states (O_2(g)_ + *e*^−^ → O_2_^−^_(ad)_) can cause electron depletion in the channel. Next, the surface roughness (due to the presence of surface defects) and impurity may cause electron scattering, leading to the limited mobility. It is still higher than other application of photodetector of oxide semiconductor materials [33-35]. This implies that it may affect the sensitivity of the photodetector. Furthermore, according to *σ* = *nqμ*, where the *σ* is the conductivity, *n* is the electron concentration, *q* is the charge of an electron, and *μ* is the mobility, the corresponding electron concentration (*n*_e_) of the InSb nanowire was estimated to be 3.6 × 10^17^ cm^−3^.

**Figure 3:**
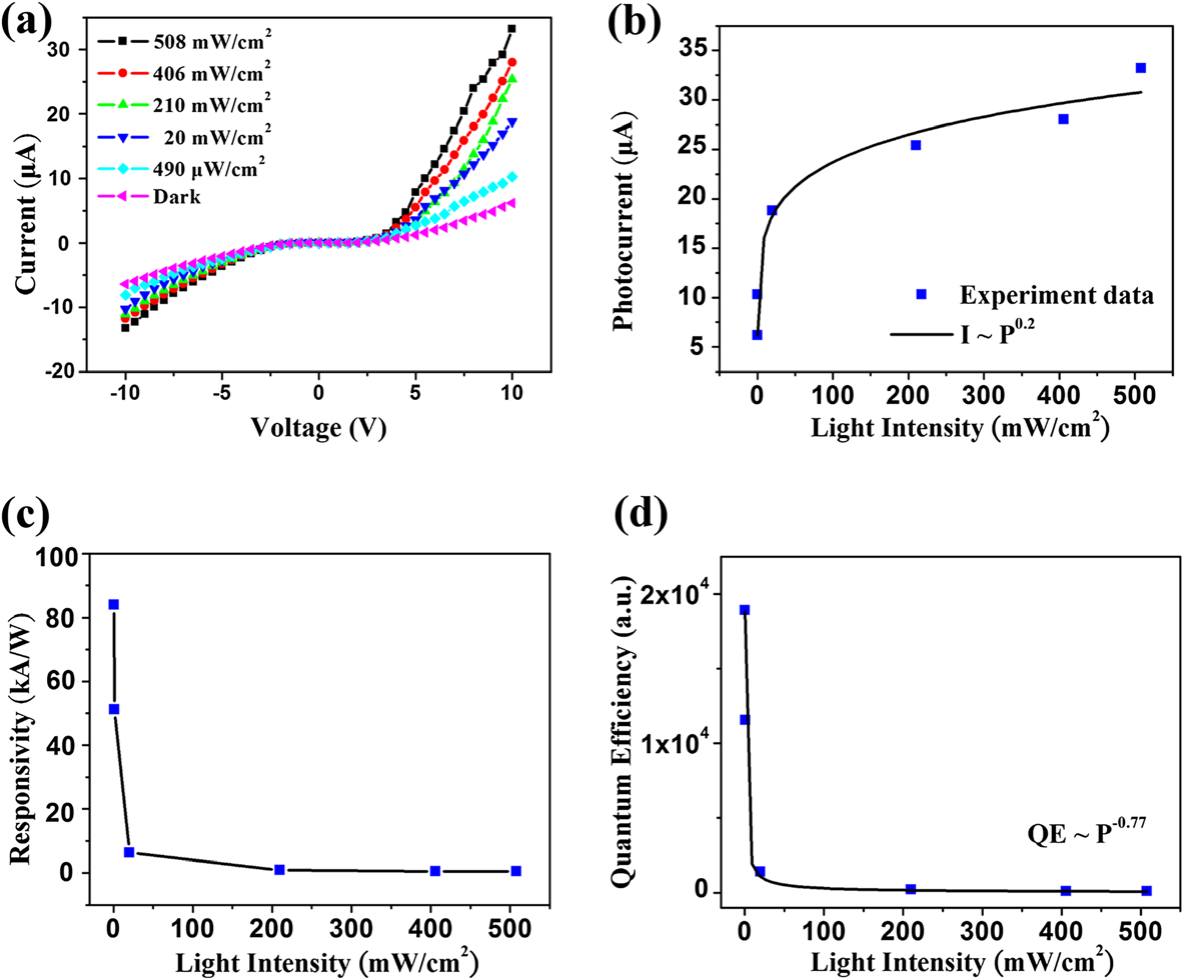
**The photoresponse properties of middle-infrared photodetector based on InSb nanowire. (a)***I*-*V* curve of an InSb nanowire under irradiation of light with different intensities. **(b)** Dependence of photocurrent on light intensity and the fitted curve using the power law. **(c)** Dependence of responsivity on light intensity. **(d)** Dependence of quantum efficiency on light intensity and the fitted curve using the power law.

To understand the photoresponse characteristics of the InSb nanowires, a single InSb nanowire was connected with the Pt Schottky contact electrodes to fabricate a nanodevice based on the M-S-M structure and measured using a Keithley 4200 system. The Pt-InSb-Pt structure constitutes a typical M-S-M photodetector. The photocurrent of the InSb nanowire is dependent on light intensity. Figure 3a shows the *I*-*V* curves of the InSb nanowire irradiated with a wavelength of 5.5 μm at different light intensities. The symmetric rectifying *I*-*V* curves exhibited two characteristics of back-to-back Schottky contacts at the two ends of the InSb nanowire. Furthermore, it shows that the conductance increases from 618.9 nS in a dark state to 3320 nS in a state of light intensity of 508 mW cm^-2^. The simultaneous increase of the photocurrent with the light intensity is consistent with the carrier generation efficiency being proportional to the absorbed photon flux. Figure 3b shows that the photocurrent dependence on light intensity can match a simple power law: *I* = *AP*^*θ*^, where *A* is a constant for a certain wavelength, and the exponent *θ* determines the response of the photocurrent to the light intensity. Fitting the curve yields *θ* = 0.2. The non-unity and a small *θ* suggest a complex process of electron–hole generation, recombination, and trapping [36]. Furthermore, the result implies the existence of numerous defects for the InSb nanowire. The existence of defects may derive from the surface vacancy, as reported in our previous work [25]. The same phenomenon had been observed in studies on CdS nanobelts [37] and CdTe nanoribbons [38]. In addition, the quantum efficiency (QE) is a critical parameter in evaluating a photosensitive device, which relates to the number of electron–hole pairs excited by one absorbed photon, and can be used to determine the efficiency of electron transport and collection by electrodes. A high QE corresponds to a high sensitivity. The QE can be expressed by the following equations [39]:

(3) $${\mathrm{QE}=N}_{e}/N_{p\;}{=\tau /t}_{\mathrm{tran}}=h{\mathrm{cR}}_{\lambda }/\mathrm{e\lambda}\;$$

(4) $$R_{\lambda }=\Delta I/\mathrm{PS}$$

where *N*_e_ is the number of electrons collected in a unit time, *N*_p_ is the number of photons absorbed in a unit time, *τ* is the carrier lifetime, *t*_tran_ is the transit time between the electrodes, and *λ* is the wavelength of irradiated light. *R*_λ_ is the spectral responsivity, defined as the photocurrent generated per unit of power of the incident light on effective areas. *ΔI* is the difference between a photocurrent and a dark current, *P* is the incident light intensity, and *S* is the area of the nanowire. For the incident light of 5.5 μm at 0.49 mW cm^−2^, *R*_λ_ is 8.4 × 10^4^ A W^−1^. This corresponds to a QE of 1.96 × 10^6^%. These high values might rival or surpass some of the reported photodetectors, such as ZnS nanowires (*R*_λ_ of approximately 1.86 A W^−1^ and QE of approximately 7.1 × 10^2^%) [40], CdTe nanoribbons (*R*_λ_ of approximately 7.8 × 10^2^ A W^−1^ and QE of approximately 2.4 × 10^5^%) [38], ZnSe nanobelts (*R*_λ_ of approximately 0.12 A W^−1^ and QE of approximately 37.2%) [10], CdS nanoribbons (*R*_λ_ of approximately 39.5 A W^−1^ and QE of approximately 1.0 × 10^4^%) [11], and WS_2_ nanotubes (*R*_λ_ of approximately 3.14 A W^−1^ and QE of approximately 615%) [41]. The *R*_λ_ dependence on the light intensity is shown in Figure 3c. The dependence of QE on the light intensity is also plotted, as shown in Figure 3d. This logarithmic plot shows that the relation of QE of approximately *P*^−0.77^ fits the power law.

This work finds that *R*_λ_ and QE decrease with increasing light intensity. The reductions of *R*_λ_ and QE are strong manifestations of a hole trap at a relatively high light intensity. Under illumination, the photogenerated holes were trapped by the oxygen ions, and the electrons contributed to the photocurrent. However, the saturation of the electron is trap at high light intensity, reducing the number of available hole traps because of the increasing recombination of photogenerated electron–hole pairs [38,42]. Furthermore, the onset of electron–hole pair recombination at a high light intensity might also contribute to the shortening of the carrier lifetime.

**Figure 4:**
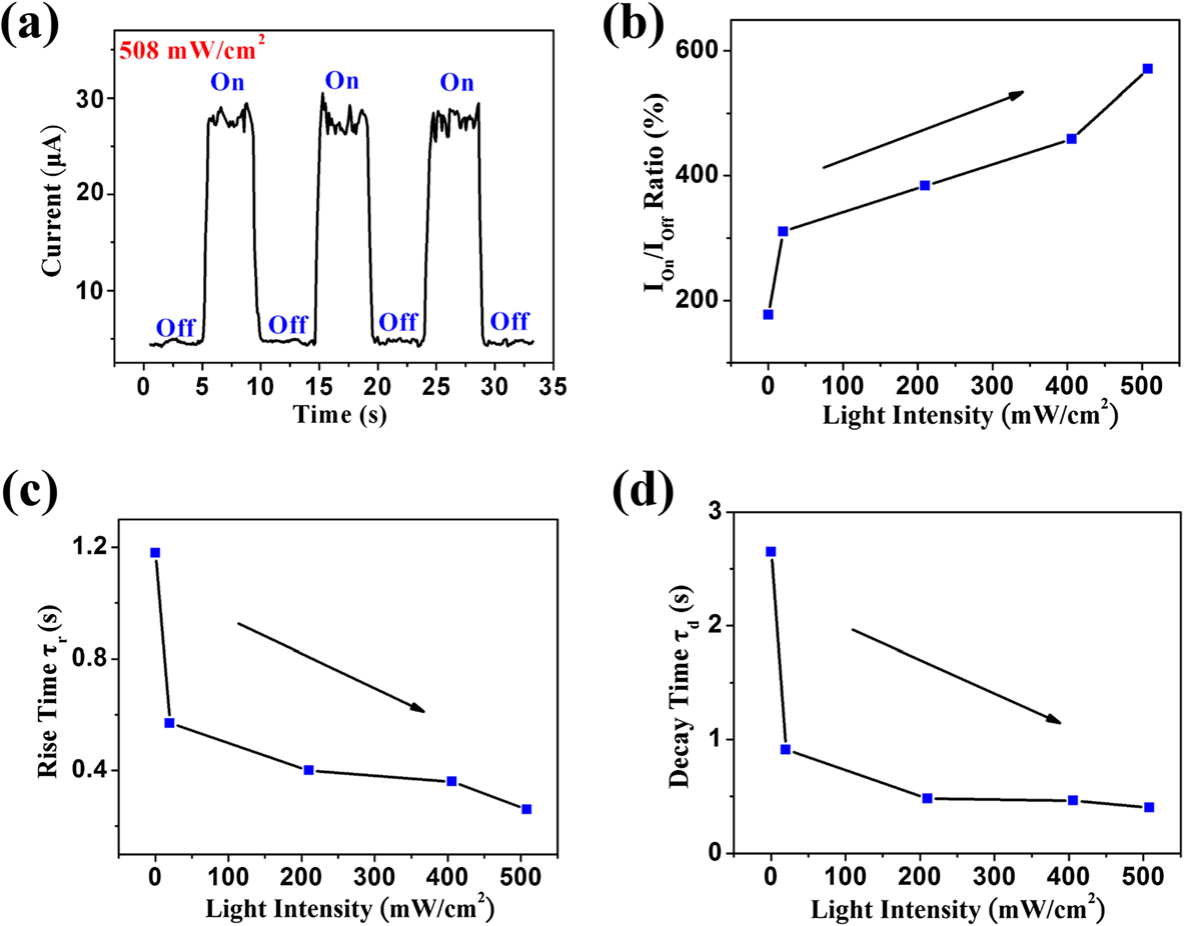
**The photocurrent properties of middle-infrared photodetector based on InSb nanowire. (a)** The photocurrent behaviors of the InSb nanowire illuminated under light intensity of 508 mW cm^−2^ as switch on and off states. **(b)***I*_on_/*I*_off_ ratio under light different intensities. **(c)** Rise and **(d)** decay of time constant at different light intensities.

The sensitivity and response speed determine whether a photodetector can feasibly perform as an optical switching device. Therefore, a fast response speed is also a crucial concern. However, the response speed is proportional to the carrier lifetime [43]. The time-dependent photoresponse of the InSb nanowire at light intensities of 508 mW cm^-2^ was measured by periodically switching on and off at a bias of 9 V, as shown in Figure 4a. The photocurrent exhibits a good, clear, and stable variation. Furthermore, the photocurrent recovered swiftly to its original value when the illumination ceased. The photocurrent-to-dark current ratio (*I*_on_/*I*_off_) increases from 177% to 571% when the light intensity increases from 0.49 to 508 mW cm^−2^, as shown in Figure 4b. Figure 4c and d illustrates the time constants for the response (rise) and the recovery (decay) edges at different light intensities, respectively. The time constants for rise and decay edges of a single cycle can be shown in the following equations [44], respectively:

(5) $$I=I_{0}\left(1-e^{{‒t/\tau }_{r}}\right)$$

(6) $$I=I_{0}e^{{‒t/\tau }_{d}}$$

where *I* and *I*_0_ are the photocurrents with and without illumination, respectively; *τ*_r_ is the time constant for the rise edge, and *τ*_d_ is the time constant for the decay edge. Both the rise and decay edges of the photocurrent match the mentioned exponential equation. The time constant *τ*_r_ decreases from 1.18 to 0.26 s when the light intensity increases from 0.49 to 508 mW cm^−2^. Furthermore, the time constant *τ*_d_ decreases from 2.65 to 0.40 s when the light intensity increases from 0.49 to 508 mW cm^−2^. In this case, both *τ*_r_ and *τ*_d_ decrease with an increasing light intensity because of the distribution of traps in the energy band of the InSb nanowires. When the light is switched on, the excess electrons and holes are generated, and subsequently, two quasi-Fermi levels (one for electrons and one for holes) are induced. When the light intensity increases, the quasi-Fermi levels for electrons and holes shift toward the conduction and valence bands, respectively, and an increasing number of traps are converted to recombination centers [5,44]. Therefore, the rise and decay times decrease significantly, and the response and recovery speeds increase. In this work, the time constants are higher than those reported elsewhere because of the defect trapping (surface vacancy) in this process. The photogenerated electrons might first fill traps to saturate them and subsequently reach the maximum number, which delays reaching a steady photocurrent. Moreover, the photogenerated electron, in returning to the valence band from the conduction, might first become trapped by the defects before reaching the valence band, which delays reaching a steady dark current [36,45]. The defect trapping can increase the carrier lifetime (enhancing QE); however, the response and recovery times also increase. Furthermore, the rise time *τ*_r_ is smaller than the decay time *τ*_d_. The long decay time can be attributed to the trapping and adsorption processes of the oxygen surface [46].

In this work, the high QE for the InSb nanowires is ascribed to the high surface-to-volume ratio and superior crystallinity of the InSb nanowires and the M-S-M structure. The high surface-to-volume ratio can significantly increase the number of hole-trap states and prolong the carrier lifetime. In the dark, oxygen molecules are adsorbed on the nanowire surface and capture free electrons (O_2(g)_ + *e*^−^ → O_2_^−^_(ad)_), and thus, the depletion layer forms near the surface, which reduces the density and mobility of the carrier. When illuminated (*hν* → *e*^−^ + *h*^+^), electron–hole pairs are generated; the holes migrate to the surface and discharge the adsorbed oxygen ions through an electron–hole recombination (*h*^+^ + O_2_^−^_(ad)_ →O_2(g)_). Unpaired electrons become major carriers that contribute to the photocurrent. This hole-trapping process significantly separates the electron–hole pairs and largely increases the carrier lifetime. [3,4,47] Meanwhile, the superior crystallinity of InSb nanowires can reduce the scattering and carrier trapping during the transport process between two electrodes, and the photocurrent rapidly reaches a steady state in both the response and the recovery stages [48].

**Figure 5:**
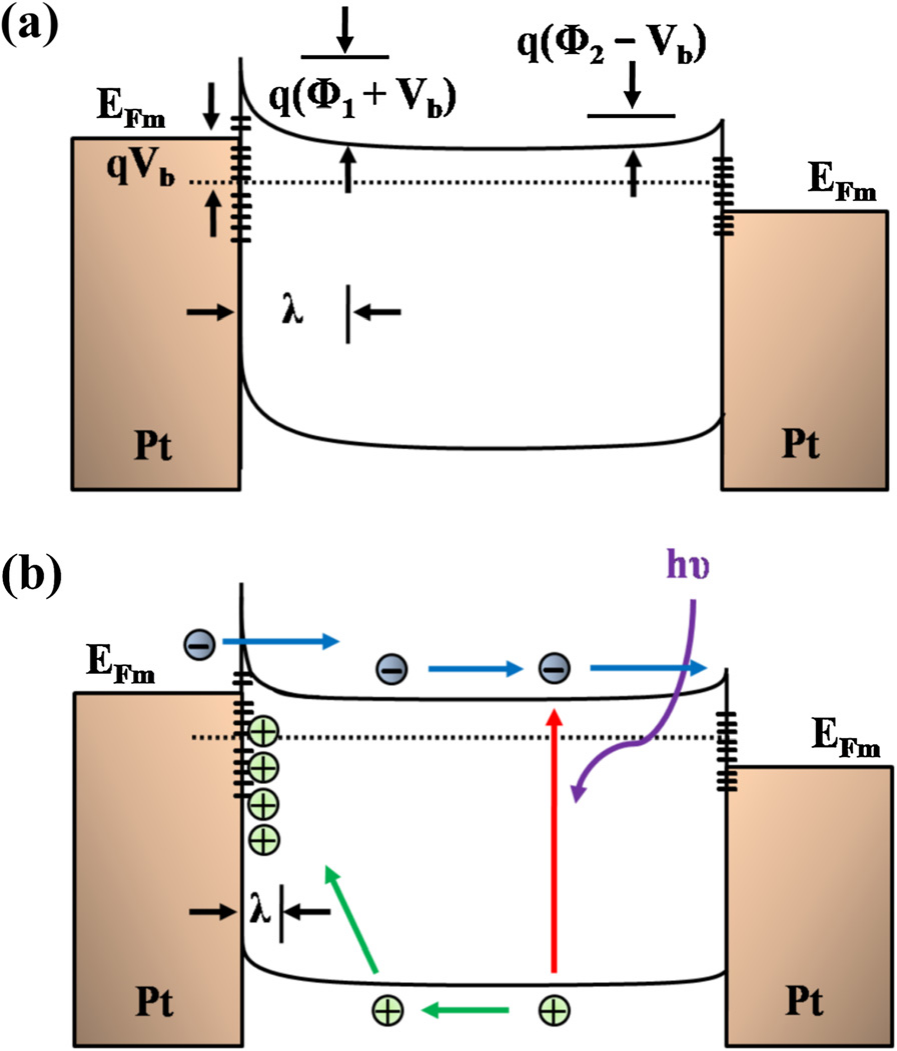
**Band diagrams of metal–semiconductor-metal structure. (a)** Dark conditions under bias *V*_b_ and **(b)** under illumination with bias *V*_b_. *Φ*_1_ and *Φ*_2_ are the Schottky barriers at the two ends. *λ* is the depletion width.

Additionally, the electron mobility may affect *t*_tran_ and enhance the QE. [36] Because *t*_tran_ = *l*/*v* and *v* = *μE* (where *l* is the electrode distance) the carrier drift velocity *v* is the product of mobility *μ* and the applied electric field, while the QE can be rewritten as QE = *τ*/*t*_tran_ = *τμE*/*l*. In this work, the mobility value of the InSb nanowire is 215.25 cm^2^ V^−1^ s^−1^, which guarantees the effective transport of the electrons between two electrodes. Finally, the M-S-M structure with back-to-back Schottky contacts can significantly enhance the photocurrent density and further increase the sensitivity of the device. The enhancement is caused by the enhanced surface band-banding effect due to the existence of the localized Schottky contact, leading to a pronounced electron–hole separation effect. Figure 5a illustrates the band diagrams of the Schottky barrier with a reverse bias in the dark. The depletion region (*λ*) near the InSb nanowire surface is formed by the surface state in the contacted region between the depletion region and the Pt electrode. In the dark, the width of the depletion region is thick, which hinders the carrier flow and, therefore, reduces the dark current. Under illumination, the photogenerated electrons and holes are attracted to lower energy sites, subsequently leading to transporting the electrons and the holes along two paths. Moreover, the separation of electrons and holes further reduces the recombination probability and significantly increases the lifetime. The holes are mostly trapped in the depletion region under a reverse bias. The redistribution of the space charge increases the positive charge density in the depletion region, thereby shrinking its width. The narrowing of the depletion region allows the electrons to tunnel in the nanowire. Contemporarily, the accumulated positive charge attracts electrons from the electrode into the nanowire, resulting in the enhancement of a current gain greater than unity and increasing the electron transport speed [49,50], as shown in Figure 5b. Furthermore, the oxygen is desorbed and reabsorbed in the interfacial region rather than over the entire surface of the nanowire. Therefore, the response and recovery time significantly decrease [51].

## Conclusion

This work demonstrated the feasibility of synthesizing single-crystal InSb nanowires using the electrochemical method at room temperature. Characteristic FET devices based on InSb nanowires have n-type conductivity because of the Sb vacancies. Meanwhile, InSb nanowires have an electron concentration of 3.6× 10^17^ cm^−3^ and an electron mobility of 215.25 cm^2^ V^−1^ s^−1^. Individual InSb nanowire was fabricated for M-IR photodetectors based on the M-S-M structure. A power-law dependence of the photocurrent on the light intensity was observed, which suggests the existence of defect states that are consistent with an n-type conductivity mechanism in the InSb nanowires. Moreover, the photodetectors exhibit good photoconductive performance, good stability and reproducibility, superior responsivity (8.4 × 10^4^ A W^−1^), and quantum efficiency (1.96 × 10^6^%). These unique properties are attributed to the high surface-to-volume ratio and superior crystallinity of InSb nanowires. In addition, the M-S-M structure can further enhance *N*_e_ (or *ΔI*) and the electron transport speed, significantly increasing the sensitivity of the photodetectors. The superior photoelectric properties of InSb nanowires are highly promising for application in high-sensitivity and high-speed nanoscale optical communication devices and photodetectors.

## Competing interests

The authors declare that they have no competing interests.

## Authors' contributions

CHK wrote the manuscript and performed all the experiments and the data analysis. SJL and JMW provided the information and organized the final version of the paper. WCC has produced the FET device. All authors read and approved the final manuscript.

## Authors' information

CHK and WCC are PhD students at National Tsing Hua University. SJL holds a professor position at National Tsing Hua University. JMW holds an associate professor position at National Tsing Hua University.
